# A Rapid RT-LAMP Assay for SARS-CoV-2 with Colorimetric Detection Assisted by a Mobile Application

**DOI:** 10.3390/diagnostics12040848

**Published:** 2022-03-29

**Authors:** María Aurora Londono-Avendano, Gerardo Libreros, Lyda Osorio, Beatriz Parra

**Affiliations:** 1 Departamento de Microbiología, Escuela de Ciencias Básicas, Facultad de Salud, Universidad del Valle, Calle 4B # 36-00, edificio 120, oficina 223/229, Cali 760043, Colombia; gerardo.libreros@correounivalle.edu.co (G.L.); beatriz.parra@correounivalle.edu.co (B.P.); 2 Escuela de Salud Pública, Facultad de Salud, Universidad del Valle, Calle 4B # 36-00, edificio 120, oficina 223/229, Cali 760043, Colombia; lyda.osorio@correounivalle.edu.co

**Keywords:** SARS-CoV-2, COVID-19, isothermal amplification, colorimetry, cell phone application

## Abstract

Loop-mediated amplification has been promoted for SARS-CoV-2 screening, however, antigen tests are preferred in low-income countries and remote zones. Poor training in molecular biology, plus the need for RNA purification or reading instruments to overcome issues of sensitivity in colorimetric detection, are some of the reasons limiting the use of this technique. In this study, nasopharyngeal swabs, aspirates and saliva were amplified in an in-house LAMP assay and subject to colorimetric detection, achieved by the naked eye and by image analysis with a mobile application. Accuracy of detection by the naked eye ranged from 61–74% but improved to 75–86% when using the application. Sensitivity of the digital approach was 81% and specificity 83%, with poor positive predictive value, and acceptable negative predictive value. Additionally to the reported effect of some transport media’s pH, the presence of mucus and warming up of reagents while setting up the reaction critically affected performance. Accuracy per type of sample was 55, 70 and 80%, for swabs, aspirates and saliva, respectively, suggesting potential to improve the test in saliva. This assay, carried out in a closed tube, reduces contamination, has few pipetting steps and requires minimal equipment. Strategies to improve performance and implications of the use this sort of colorimetric LAMP for massive testing are discussed.

## 1. Introduction

Loop-mediated amplification with reverse transcription (RT-LAMP) has become a popular alternative to real-time polymerase chain reaction (qRT-PCR) in SARS-CoV-2 testing. One year after the World Health Organization declared a pandemic of COVID-19 [1], many protocols using this isothermal technique have been described in peer-reviewed journals. In many cases, detection limits of LAMP are comparable to PCR [2,3,4]. This, combined with a lower cost of amplification enzymes, has been the rationale behind the proliferation of these assays. LAMP is also one of the strategies for target enrichment in microfluidic sensors [5,6,7,8] and other devices being developed for point of care testing of this virus [9,10,11,12].

LAMP assays for SARS-CoV-2 surged in developed economies such as Japan, Korea, China, the United States and, to a lesser extent, the United Kingdom and the European community; among these, a few have emergency use approval from the Food and Drug Administration [13], and the European Centre for Disease Prevention and Control [14]. Early publications presented proof of principle [4,7,15,16,17,18,19,20,21,22,23,24], but those reporting accuracy in hundreds of samples increased during the second epidemic wave [6,25,26,27,28,29,30,31,32,33,34,35,36,37,38]. Although these authors indicate that RT-LAMP might constitute an affordable alternative for mass screening and point of care testing in areas where resources are limited, there are many reasons why this technique is not outcompeting antigen-based tests in middle- and low-income countries. Sensitivity of RT-LAMP in detecting SARS-CoV-2 ranges from 70–100%, depending on many factors [2,28,31,32,39,40]. At least two main categories of sample processing, RNA isolation and RNA release, plus three options for detection, need to be compared. Assays achieving agreement superior to 95% with qRT-PCR are those with RNA purification plus fluorometric [27,38,41,42], turbidimetric [38] or colorimetric detection [25,26,32,33,43,44]. RNA purification incurs a higher cost and longer process, pushing these approaches outside the scope of a rapid test. RNA release, although cheaper, faster and easier to implement, results in lower sensitivity [6,25,29,34,36,43,45,46,47,48].

A few LAMP protocols combining RNA release with colorimetric detection achieve accuracy superior to 80%. Among these, Dao Thi and co-workers [33] processed samples by boiling and detected the color turnout with microplate readers; Schellenberg and Yang, with their collaborators, relied on the naked eye but emphasize that performance is strongly influenced by pH in the sample, which depends on both the type of sample and the transport media [3,37,49]. Implementing LAMP assays by typical reactions in which pipetting of primers, enzyme and other ingredients is needed are time-consuming, at risk of contamination and discouraging for personnel not habituated to techniques in molecular biology; in spite of this disadvantage, LAMP is considered the easiest for rapid training in molecular identification of microbes [50,51]. It is noteworthy that enzymes for RT-LAMP are distributed globally by few providers, representing another limitation for low-income countries, not to mention the probabilities of a shortage in supplies [2,20]. To make colorimetric amplification deployable in primary care centers typical of rural Colombia, we evaluated an RT-LAMP assay that: (1) adopts fast sample processing and reduces pipetting steps, (2) uses closed tubes to minimize cross-contamination and (3) assists/replaces naked-eye detection with affordable software. Accuracy of the approach resembles that of commercial assays already introduced to low-income countries [30,35,52,53], but strategies to improve the performance of colorimetric LAMP and troubleshooting are discussed.

## 2. Materials and Methods

### 2.1. Origin of Samples

Nasopharyngeal swab (NPS), nasopharyngeal aspirate (NPA) or saliva (SA) stored in the repository of biological samples of the Laboratorio de Diagnóstico de Agentes Biológicos (LDAB) at the College of Health of the Universidad del Valle were used with authorization from the institutional human research ethics committee. LDAB facilities hold authorization from the Colombian National Institute of Health to perform SARS-CoV-2 testing by real-time PCR in NPSs and NPAs, which arrive in diverse transport media. Saliva was collected without transport media, under two research protocols approved by the institutional ethics committee.

### 2.2. Real-Time RT-PCR

Amplification was performed in RT-7500 or QuantStudio 5 thermal cyclers (Life Technologies, Carlsbad, CA, USA) following United States CDC recommendations [54]. Reactions contained a total final volume of 20 µL with 5 µL of template RNA, 500 nM of primer/probe mix and 1X SuperScript III One-Step RT-PCR System with Platinum Taq DNA polymerase (Life Technologies Corporation, Carlsbad, CA, USA). Amplification parameters were 50 °C-30 min; 95 °C-2 min; 45 cycles of: 95 °C-15 s, 55 °C-30 s. A cycle threshold (Ct) value of 42, or lower, for the N1 gene was considered to be positive for detection, while samples giving undetectable Ct for N1 but detectable N2 were considered undefined; samples giving RNAseP Ct lower than 42 and undetectable values for of N1 or N2 were considered negative.

### 2.3. Primers and Initial Conditions for LAMP

Two sets of primers (“Primers set A” [23] and “Primers set B” [55]) were initially evaluated. LAMP reactions were performed in 0.2 mL tubes containing molecular grade water, 12.5 µL of WarmStart master mix (New England Biolabs, Ipswich, MA, USA), plus one of three different preparations of each primer set, then incubated at 65 °C for 30 and 40 min and inactivated at 85 °C. Results were detected by the naked eye with three possible outcomes: negative (sample remains pink), faint (hard to report a change, reaction turns orangish) and positive (sample turns yellow); correspondence between color changes and amplification was evaluated by gel electrophoresis with four possible outcomes: positive (bands with the expected size pattern), negative (only primer bands detectable), inefficient amplification (smear within the expected sizes) and reduced specificity (bands with an abnormal size pattern). Under these conditions, “Primers set A” yielded reduced specificity (data not shown) and therefore findings correspond to “Primers set B” from here on.

Primers set B: nts 13,434–13,636, ORF1ab

BF3: TGCTTCAGTCAGCTGATG

BB3: TTAAATTGTCATCTTCGTCCTT

BFIP: TCAGTACTAGTGCCTGTGCCCACAATCGTTTTTAAACGGGT

BBIP: TCGTATACAGGGCTTTTGACATCTATCTTGGAAGCGACAACAA

BLoopF: CTGCACTTACACCGCAA

BLoopB: GTAGCTGGTTTTGCTAAATTCC

### 2.4. Mobile-Assisted Visualization

To solve cases where the naked eye finds undefined results, the mobile version of SpotXel (GmbH, Germersheim, Germany) was used [56]. Reaction tubes were photographed before and after incubation at 65 °C; for this, tubes were accommodated in horizontal racks in a set up that positions the cell phone at a predefined height and illumination. Photographs were later used to obtain values of color intensity, using the microarray option of SpotXel (referred as “yellow intensity” from here on) with the grid adjusted to one square per tube; recorded values were both exported in tables and stored as screen shots. Samples yielding ≥ 1.0-fold change in the value of yellow intensity were considered positive.

### 2.5. Optimization to Avoid RNA Purification

Four strategies for sample processing were tested prior to evaluating the sensitivity and specificity of a final protocol with “Primers set B”. RNA purification was performed with the *AccuPrep*^®^ viral RNA extraction kit (Bioneer Corporation, Daejeon, Korea) following the manufacturer’s instructions. Fast processing was carried out by mixing 50 µL of the sample 1:1 in PCR*opsis* Reagent RVD (Entopsis, Miami, FL, USA) and boiling at 95 °C for five minutes, as indicated by the manufacturers. Inexpensive processing was carried out by diluting 20 µL of the sample 1:1 in PBS or DEPC water and boiling at 95 °C for five minutes. The outcome was evaluated as the percentage of positive and negative agreement by gel electrophoresis, visual detection and mobile application as described above.

### 2.6. Rapid LAMP Strategy

“Primers set B” were prepared to final concentrations and stored in stocks of 24 reactions at −20 °C; parallel stocks of 24 tubes containing 20 µL of “lysis buffer” (DEPC water, Sigma-Aldrich, Darmstadt, Germany) were stored at room temperature. For sample processing, one tube of primers and one tube of lysis buffer per sample, plus positive and negative controls, were used in the next steps: (i) in the sample manipulation area, using a microbiological cabinet, 20 µL of the sample was added to “lysis buffer”, then heated at 95 °C for five minutes in a thermal block; (ii) in the clean area, 12.5 µL of WarmStart enzymes was added to the prepared primers; (iii) back in the sample manipulation area, 2.0 µL of boiled sample was added to the primers + enzyme mix, mixed thoroughly and incubated for 40 min at 65 °C in a thermal block. Results of visual and application-assisted detection were recorded as described above.

### 2.7. Detection Limit of the Rapid Assay

Detection limits were established by spiking NSP, NPA and saliva. Briefly, 50 µL of total RNA purified from a positive sample carrying 871.63 × 10^6^ copies/µL of SARS-CoV-2 genetic material (measured with a curve of known amounts of synthetic positive control) was added to 450 µL of NSP, NPA or saliva previously established to have undetectable amounts of viral RNA. Serial dilutions were performed and aliquots from eight preps were subject to RNA purification and qRT-PCR to obtain a copy number, and to a rapid protocol to identify the range of detection. The outcomes were established by the visual and application-assisted methodologies described above.

### 2.8. Cross-Reactivity

Nasopharyngeal aspirates positive for eight of the most frequent respiratory viruses in Colombia (adenovirus, influenza A and B, respiratory syncytial virus, metapneumovirus and parainfluenza viruses 1, 2 and 3) and a nasopharyngeal swab containing human coronavirus OC43 were tested with the rapid LAMP procedure. Results were recorded by visual detection and application-assisted methodologies, as previously described.

### 2.9. Performance of the Rapid LAMP Assay

A total of 208 samples comprising 86 NPS, 62 NPA and 60 saliva samples were tested. Tubes were photographed before and after incubation at 65 °C for 40 min and results of visual observation and values of image analysis were tabulated. Agreement with qRT-PCR, sensitivity and other statistics of performance of the visual and the mobile-assisted detection approaches were calculated with SPP, and plotted in R (v.3.5.1, R Foundation for Statistical Computing, Vienna, Austria) using ggplot2. RT-qPCR experiments were analyzed using QuantStudio Design and Analysis Software (v.2.3.3, ThermoFisher, Waltham, MA, USA), or exported for analysis in R software.

## 3. Results

### 3.1. Setting Up the Conditions

**Figure 1:**
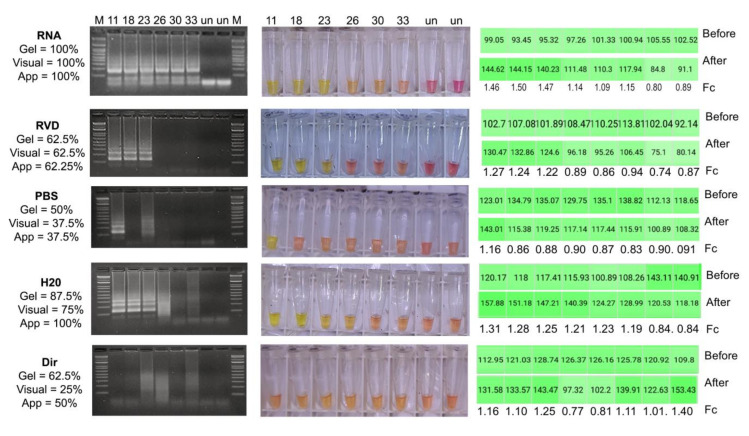
Agreement of sample pre-processing strategies. Percentages in each strategy are values of total agreement, calculated based on results for eight NPSs and four NPAs with Ct from 11–33 plus two negatives of each type of sample (labeled un); for esthetic reasons, only half of the pictures are shown. For all gels: smears were counted as positives, M = 100 pb ladder, numbers 11, 18, 23, 26, 30, 33 = Ct of the sample. For tubes for visual detection: “orangish” was taken as negative, numbers 11, 18, 23, 26, 30, 33 = Ct of the sample. For the application (App): order is for tubes shown for visual detection (Ct 11, 18, 23, 26, 30, 33, un, un), Fc (fold change) = after/before; if Fc > 1.0, the sample was classified as positive.

The colorimetric assay was first tested under the ideal conditions, i.e., with purified RNA, which gave positive outcomes observable down to Ct from 30–33 (Figure 1). Those Ct values correspond to ~621–97 copies of SARS-CoV-2 genome/µL in the real-time protocol used. At this point, the RT-LAMP assay did not generate faint results and the colorimetric detection coincided with electrophoresis in all repeats. Visual detection was feasible with RVD in those samples with Ct lower than 23, reaching an agreement of 62.5% in the three repeats. Pre-processing with PBS and direct addition of the sample gave the lowest percentages of agreement, which were also inconsistent between gel, color turnout and the application; for these treatments, the electrophoresis indicated null or inefficient amplification even in samples with high viral loads. The use of DEPC water allowed amplification for samples with Ct up to 23 that matched electrophoresis, visual outcome and fold change in yellow intensity; if inefficient amplification is counted as positive by contrast to the lack of a smear in negative samples, the accuracy of this treatment in electrophoretic detection is 87.5%. DEPC processed samples with Ct lower than 23 had negative visual outcomes but the changes in color intensity were enough to be classified as positive by the application, however, fold change values in these samples lacked a clear match to viral load, probably because samples consisted of randomly distributed 60% NPS and 40% NPA. Overall, boiling the sample in water gave closer results to RNA purification, with a total agreement of 75% in the visual detection and 100% when using SpotXel. It is worth noting that with the use of purified RNA or pre-processing in RVD, the reaction starts at a higher pH compared to the other strategies; this is a factor against colorimetric detection that will be addressed below.

### 3.2. Detection Limit of a Selected Strategy

The rapid, mobile-assisted protocol correctly detected positives when testing samples with 22,594, 112,814 and 88,682 copies/µL of SARS-CoV-2 RNA in NPSs, NPAs and saliva, respectively. These values correspond to Cts from 21–25, in agreement with the preliminary findings presented above for H20 + boiling. Detection was still obtained in other dilutions but repeatability dropped for both visual and application-based detection (Table 1).

### 3.3. Cross-Reactivity

The assay did not produce positive results when used with samples known to have other respiratory viruses (Table 2). Most samples, however, were nasopharyngeal aspirates and this finding may not translate to other types of samples.

### 3.4. Performance of the Visual Approach

**Figure 2:**
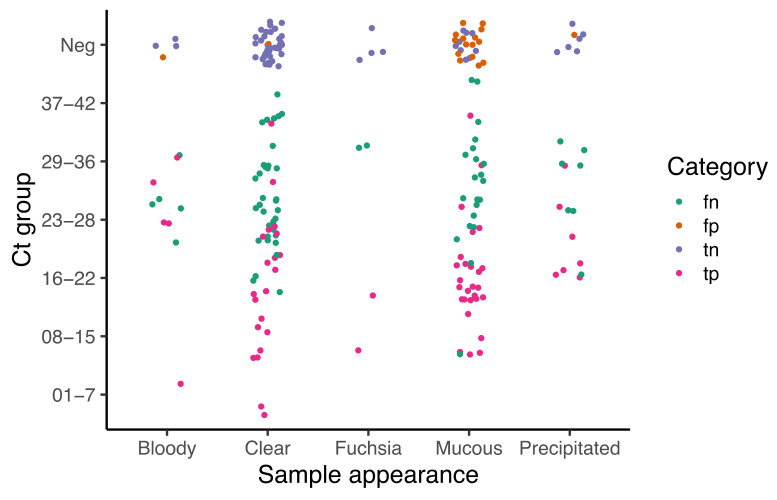
Effect of sample quality on visual detection. Categories correspond to: tp = true positives, tn = true negatives, fp = false positives, qRT-PCR negatives that turned yellow in LAMP, fn = false negatives, qRT-PCR positives that gave faint or negative visual outcomes. Percentage of agreement with qRT-PCR is 57.1, 62.4, 75.0, 50.0 and 62.0, for bloody, clear, fuchsia, mucus and precipitated conditions, respectively.

This approach gave an accuracy of 68%, with an elevated number of false negatives (summarized in Table 3). Global agreement with qRT-PCR, calculated as global kappa index, is fair (0.34), however, after analyzing data by type of sample, better agreement is observed with saliva (0.44, 0.24–0.66), followed by NPA (0.03, −0.20–0.26), and NPS (0.02, 0.16–0.20) (see Supplementary Table S1). The high number of faint results is associated with mucus in the sample and the viral load (see Figure 2). Those samples classified as mucus resulted more often in false positives than those with any other type of appearance; there are false negatives in all categories of sample quality but they concentrate in groups with Ct values above 23 (Figure 2), which again represents the characteristic sensitivity identified during optimization. Interestingly, bloody samples and those collected in transport media containing pH indicator (fuchsia), which remain reddish after boiling, did not tend to give false negatives.

### 3.5. Performance of Assay with Mobile Application

**Figure 3:**
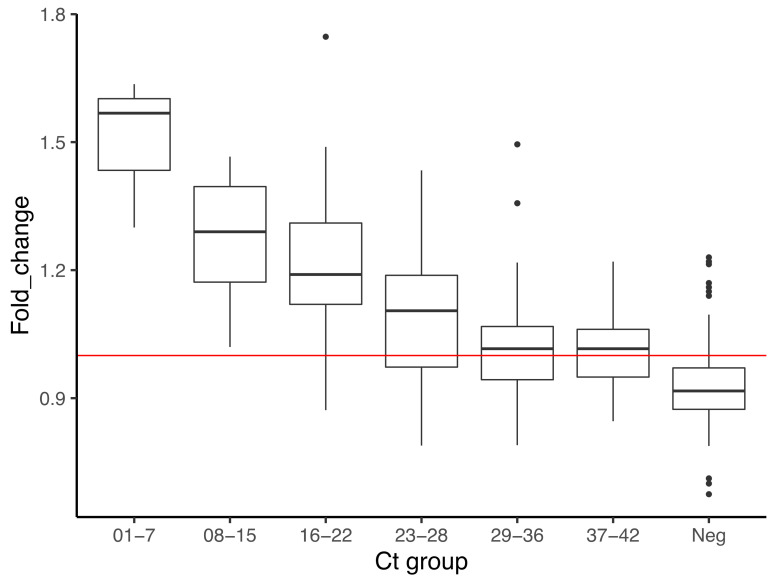
Relation between fold change and viral load. Y-axis indicates fold change values for a total of 208 samples, with the horizontal line corresponding to the threshold established. Boxes contain average and standard deviation of the fold change per Ct group, where Neg corresponds to the group with undetectable RNA of SARS-CoV-2 by qRT-PCR. The red line marks a fold change = 1.0.

The accuracy of this approach is nearly 81%, which is below the 85% threshold that the Colombian Institute of Health has recommended for SARS-CoV-2 diagnostics (Table 3). There was a moderate agreement with the qRT-PCR protocol (kappa index = 0.55) which, as discussed below, is related not only to detection limits but to technical issues and a considerable number of false positives. Performance was slightly better in saliva (79.5% of accuracy) compared to NPS and NPA (~60% and 70% accuracy, respectively), which suggests a potential to improve detection in saliva after further optimizations of the assays. Fold change has clear differences in the average between true positives and true negatives (1.20 ± 0.16 vs. 0.89 ± 0.07, respectively) (Table 3). This represents changes of 22–32% in yellow intensity, which, as illustrated in Figure 3, has a relation to viral load, but there is no linearity. Variation in fold change was high in negatives, but also in intermediate samples with Ct from 16–28; this also occurred in data from the limit of detection (data no shown), and translates into more false positives in these groups compared to those of Ct > 28.

**Figure 4:**
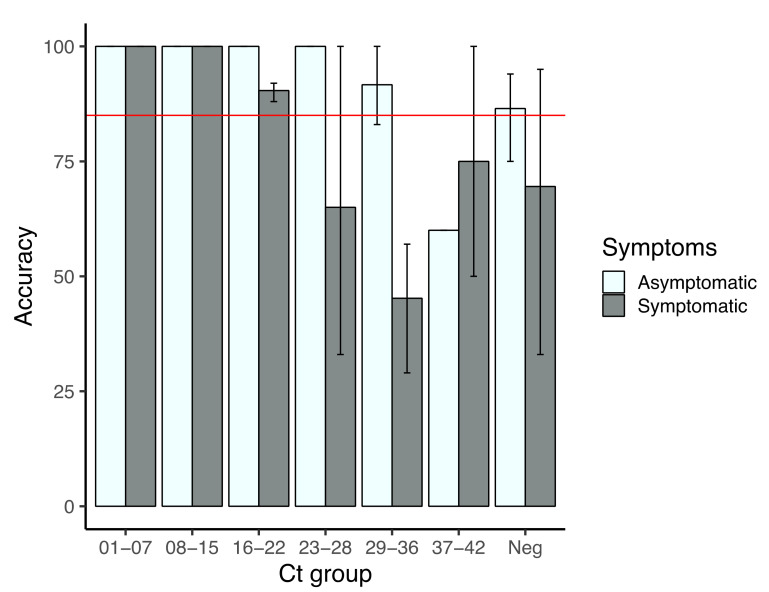
Performance of the application-mediated detection among symptomatic and asymptomatic patients. Accuracy = tp + tn/total of symptomatic or asymptomatic in each Ct group; standard deviation was established based in the calculated accuracy per type of sample in each group. The red line marks a 85% of accuracy, required in Colombia for SARS-CoV-2 diagnostic tests.

Figure 4 shows the performance of the imaging approach in terms of viral load and its potential use for SARS-CoV-2 screening. In both symptomatic and asymptomatic patients, accuracy of the assay is higher than 85% for those samples with Cts below 23, however, those with lower viral loads gave less accurate results, especially in symptomatic patients. As indicated by the standard deviations, performance varied among types of samples, with a higher reduction in the case of NPS, in which performance is from 60–50% above Ct 23; in NPA, accuracy diminished below 85% above Ct 29, whereas in saliva accuracy dropped below 50% in Ct groups 23–28 and 29–36 but increased again in Ct group 37–42 (Supplementary Figure S1).

## 4. Discussion

The detection limit with purified RNA was at some point between 621 and 97 copies of viral RNA/µL (Figure 1), but between ~112,000 and ~22,000 copies with the application. Similar to other RT-LAMP assays for SARS-CoV-2, boiling the samples in water was suitable to avoid RNA purification [3,37,49], however, when used in the visual detection mode, this assay is inaccurate; with the mobile application, the assay’s accuracy reaches up to 81%, resembling that of some commercial tests with reduced performance already introduced to low-income countries [30,35,52,53]. There is a sort of recovery in performance in samples with Ct above 29 in NPS and saliva samples, in both the visual (data not shown) and application approach (Supplementary Figure S1); the finding of a similar trend in the detection limit assays suggests a behavior of the analyte, which would explain the lack of linearity in values of fold change described in Figure 3; perhaps other mathematical approaches to transform data would adjust better to this behavior.

In addition to those previously reported, other factors that affect the performance of colorimetric LAMPs were identified. Those samples collected in sodium azide were found unfit for LAMP (data not shown), whereas those containing excessive mucus are very likely to give false positives. Other authors have shown that saliva samples have a decreased sensitivity in qRT-PCR [57] and LAMP [58,59] due to variable pH, which affects the RNA purification protocol and likely amplification, resulting in quantification that deviated from other types of samples. Mucus samples also represent difficulties for qRT-PCR which are solved by diluting and/or modifying the RNA purification methodology; here, the LAMP assays for mucus samples were carried out with material already diluted in UTM for qRT-PCR, therefore other strategies are needed to overcome this issue.

Initial intensity of pink as a factor influencing performance of both visual and application-assisted detection was left undocumented, but it is worthy of discussion. Reactions processed with purified RNA and RVD started with the deepest pink color if compared to those where the sample was processed by other strategies. This, although related to the pH of the respective buffers, seems to also depend on the sensitivity of phenol red to environmental conditions. As noticed by Dao Thi and co-workers [33], commercial reagents for colorimetric LAMP based on phenol red cannot be prepared in advance because they acidify with long exposure to air. We found that keeping the reaction mix on ice and tubes closed while adding other samples helps to maintain pink uniformity; this was incorporated as a routine procedure after optimization.

Accuracy of the mobile-assisted approach was similar to other tests of colorimetric RT-LAMP plus boiling in nasopharyngeal swabs, detecting either the N gene [33] or the E gene [49]. These findings suggests that abundance and secondary structures of the target, in this case the ORF1ab overlap, have low influence on performance. More interestingly, saliva performed better than other types of samples and has potential to be improved. Yang and collaborators [3] optimized colorimetric RT-LAMP in saliva by using a pH stabilizer with NaOH and primers for the E gene. Based on the standard deviation of fold change (Table 4), it seems that aspirates and swabs exhibit a less flexible relation of color turnout to viral load compared to saliva, likely because they are often collected in buffered solutions. This is supported by the homogeneity of fold change among positive samples when using RVD as a pH stabilizer (Figure 1). Although stabilizing saliva is acceptable for qualitative detection, it might tamper with a semi-quantitative approach because the relation of yellow intensity to viral load appears to be more linear in non-buffered samples.

Double photographing of the tubes to obtain the fold change is the main disadvantage of the application-based approach. It is time-consuming, requires uniformity of light and the same equipment to acquire both images; after identifying other factors that influence the behavior of the analyte, the inclusion of a curve in each experiment might be enough to sort out negatives and positives; setting up cutoff values for yellow intensity after incubation seems viable with a cutoff of 100, although the correlation between yellow intensity after incubation and Ct is less direct than Ct vs. fold change (Supplementary Figure S2). Although reading plates are more likely to be cross-contaminated than closed tubes in the scenarios imagined for this assay, the approach cannot be discarded for low-income countries. While regular polystyrene plates do not resist incubation at 65 °C, this assay, if transferred to ELISA reading systems in sealed polypropylene plates, might allow not only a more uniform environment with respect to light source, but also the ability to test more samples simultaneously; if the software has a similar relationship between yellow intensity and viral load in plates with a transparent seal, the assay could be performed without a need to purchase equipment, but issues in reading uniformity might still need local solutions.

## 5. Conclusions

This assay did not reach national standards to be used as a diagnostic test for SARS-CoV-2 in Colombia, but was able to identify symptomatic and asymptomatic persons with high viral loads in all kinds of samples tested. In comparison, commercial antigen-based tests, as reviewed by Dinnes et al. (2021) [60], have a sensitivity of 72–79.0% in symptomatic and 51–74% in asymptomatic patients, with major limitations in those patients sampled more than ten days after having acquired the virus. Although viral loads do not match symptomatology by being influenced by timing and expression of sub-genomic RNAs, high viral loads correlate with infectivity [61,62] and have been linked to index cases and super-spreading events [37,63]. Although viral loads are influenced by timing, they do not match symptomatology and are associated with expression of sub-genomic RNAs, high viral load correlates with infectivity [61,62] and have been linked to index cases and super-spreading events [37,63]. The Abbott ID Now assay and other low-sensitivity but widely marketed SARS-CoV-2 tests avoid the use of microbiological cabinets by using azides or NaOH during sample collection. This LAMP assay is still subject to laboratory infrastructure, but, similar to them, can easily spot individuals with high viral loads, the ones more likely to transmit the virus. Low-sensitivity tests, initially intended for point of care diagnosis, are being widely used to facilitate the return of employees who have entered quarantine and for routinely testing personnel in hospitals and schools. Implementing sample self-collection and inactivation protocols might allow the use of this assay in laboratory-free facilities, which is more likely to be achieved if sampling saliva.

The set of primers used in this study was designed to amplify the overlap between ORF1a and ORF1b genes, implying that the true positives identified are patients who actually harbor full genomes of SARS-CoV-2. In consequence, this strategy serves for detecting potential super-spreaders and its use in massive screening can have a direct impact on reducing transmission. Further studies to correlate day of contagion and symptom onset with performance of the test might reveal its suitability for early diagnostics and decision making in clinical or epidemiological scenarios.

Thus far, in-house RT-LAMP for detection of SARS-CoV2 in Latin America has been validated in Peru [64,65]. This is the first attempt to develop a local protocol and achieve efficient detection with minimal cost in Colombia. Although this assay does not meet all the affordable, specific, sensitive, user-friendly, rapid, equipment-free and deliverable to the user (ASSURED) guidelines for low-resource settings proposed by the World Health Organization [60], it has advantages in the context of the COVID-19 pandemic. A test with reduced sensitivity generates false negatives which can be compensated by re-testing, whereas if specificity is reduced, the frequent finding of false positives does not affect transmissibility but impacts the economy. Based on our results, an assay for saliva is more likely to meet the ASSURED criteria or to be used combined with other inexpensive strategies because it has the highest sensitivity and its sampling has the lower cost.

## Figures and Tables

**Table 1:** Detection limit of the rapid LAMP assay.

NPS			NPA			SAL		
Estimated N^o^ Copies	Positives Visual	Positives SpotXel	Estimated N^o^ Copies	Positives Visual	Positives SpotXel	Estimated N^o^ Copies	Positives Visual	Positives SpotXel
564,849	4/4	4/4	558,801	4/4	4/4	582,730	4/4	4/4
48,810	4/4	4/4	112,814	4/4	4/4	88,682	3/4	4/4
22,594	4/4	4/4	22,563	2/4	3/4	23,309	0/4	2/4
7001	1/4	2/4	10,827	2/4	2/4	7906	1/4	2/4
621	2/4	2/4	1381	3/4	3/4	933	0/4	1/4
181	1/4	1/4	179	3/4	2/4	186	1/4	2/4
36	0/4	2/4	36	1/4	3/4	37	0/4	0/4
0.0	0/4	0/0	0.0	0/4	0/4	0.0	0/4	0/4

**Table 2:** Cross-reactivity.

Virus	No. Samples Tested *	Positives, Visual	Positives, SpotXel
AdV	4	0/4	0/4
InfA	3	0/3	0/3
InfB	2	0/2	0/2
hMPNV	3	0/3	0/3
Parainf (1–3)	6	0/6	0/6
RSV	4	0/4	0/4
HCoV-OC43	1	0/1	0/1

* Each sample was processed twice in the fast assay, but HCoV-OC43 was also evaluated with purified RNA.

**Table 3:** Estimated performance of visual detection.

		RT-LAMP Visual			
		Positives	Faint	Negatives	Total
**qRT-PCR CDC**	Positives	62	35	34	**131**
	Faint	0	0	0	**0**
	Negatives	4	14	59	**77**
	**Total**	**66**	**49**	**93**	**208**
Accuracy *		67.8 (61.03–74.1)			
Kappa index *		0.34 (0.26–0.42)			

* Faint results were assigned to false positives or false negatives when calculating.

**Table 4:** Performance of the assay with application-assisted detection.

	Global qPCR (+) = 131 qPCR (−) = 77	NPS qPCR (+) = 61 qPCR (−) = 25	NPA qPCR (+) = 37 qPCR (−) = 25	Saliva qPCR (+) = 33 qPCR (−) = 27
**Accuracy**	80.6 (74.6–85.8)	54.8 (43.7–65.6)	69.8 (56.8–80.8)	79.5 (67.1–88.8)
**Sensitivity**	74.8 (66.5–82.0)	42.62 (30.0–55.9)	46.0 (29.5–63.1)	57.6 (39.2–74.5)
**Specificity**	83.1 (72.9–90.7)	60.0 (38.7–78.9)	80.0 (59.3–93.2)	88.9( 70.8–97.7)
**Positive predictive value**	65.51 (53.4–75.9)	31.4 (20.7–44.5)	49.6 (29.4–69.9)	68.9 (42.4–87.0)
**Negative predictive value**	88.5 (84.9–91.3)	70.9 (62.4–78.2)	77.5 (70.8–83.1)	83.0 (76.3–88.1)
**Fold change** (average ± sd)	tp = 1.20 ± 0.16	tp = 1.17 ± 0.14	tp = 1.22 ± 0.17	tp = 1.25 ± 0.15
	tn = 0.89 ± 0.07	tn = 0.91 ± 0.04	tn = 0.91 ± 0.05	tn = 0.85 ± 0.09
	fp = 1.13 ± 0.07	fp = 1.10 ± 0.07	fp = 1.01 ± 0.01	fp = 1.16 ± 0.05
	fn = 0.92 ± 0.06	fn = 0.94 ± 0.04	fn = 0.90 ± 0.04	fn = 0.90 ± 0.08

## Supplementary Materials

The following supporting information can be downloaded at: https://www.mdpi.com/article/10.3390/diagnostics12040848/s1, Table S1: Performance of visual detection per type of sample. Figure S1: Performance of the mobile-assisted detection approach by type of sample. Figure S2: Relation between yellow intensity and viral load after incubation.
