# Supplementary material for: A Rapid RT-LAMP Assay for SARS-CoV-2 with Colorimetric Detection Assisted by a Mobile Application
Source: Diagnostics (Basel). 2022 Mar 29;12(4):848. doi: 10.3390/diagnostics12040848 (PMC9032071; doi:10.3390/diagnostics12040848)

A rapid RT-LAMP assay for SARS-CoV-2 with colorimetric detection assisted by a mobile application

María Aurora Londono-Avendano[1*], Gerardo Libreros[1], Lyda Osorio[2], Beatriz Parra[1]

Table S1. Performance of visualized detection per type of sample.

	**Nasopharyngeal swab** qPCR positives=61 qPCR negatives=25	**Nasopharyngeal aspirate** qPCR positives=37 qPCR negatives=25	**Saliva** qPCR positives=33 qPCR negatives=27
**Accuracy**	54.8 (43.7-65.6)	69.8 (56.8-80.8)	79.5 (67.1-88.8)
**Sensitivity**	42.62 (30.0-55.9)	46.0 (29.5-63.1)	57.6 (39.2-74.5)
**Specificity**	60.0 (38.7-78.9)	80.0 (59.3-93.2)	88.9 (70.8-97.7)
**Pos. predictive value**	31.4 (20.7-44.5)	49.6 (29.4-69.9)	68.9 (42.4-87.0)
**Neg. predictive value**	70.9 (62.4-78.2)	77.5 (70.8-83.1)	83.0 (76.3-88.1)
**Cohen's Kappa index**	0.020 (-0.16-0.20)	0.031 (-0.20-0.26)	0.448 (0.240 - 0.656)

Calculated by assigning faint results in false positives and false negatives.

Figure S1. Performance of the mobile assisted detection approach by type of sample. NPA= nasopharyngeal, NPS= nasopharyngeal swabs, SAL= saliva. Accuracy is calculated as true positives + true negatives/total of each type of sample, in each Ct group. The red line, in 85, demarks the performance required by the test to be suitable for SARS-CoV-2 testing.

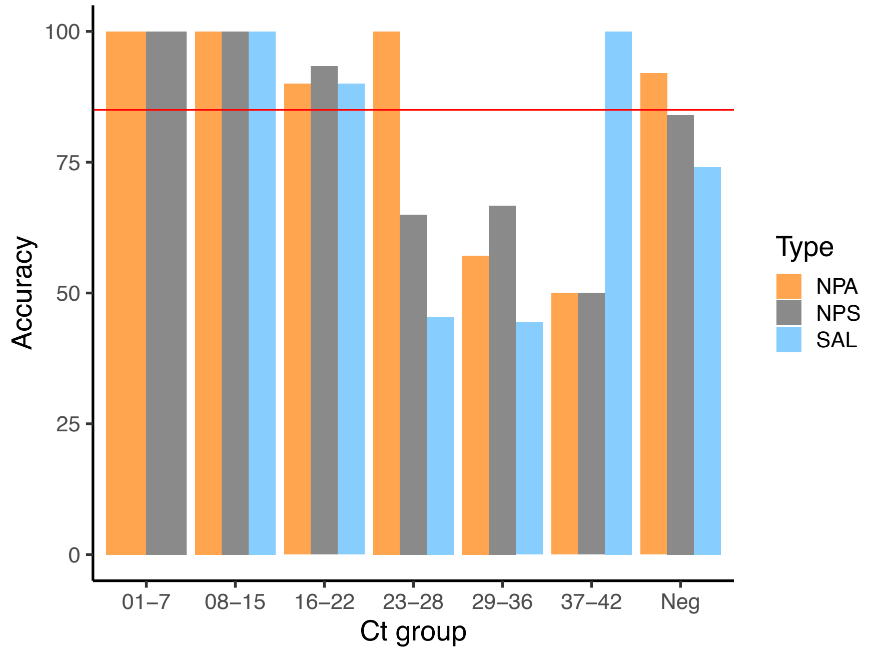

Figure S2. Relation between yellow intensity and viral load after incubation. Axis Y indicates color intensity values for total 208 samples, with the horizontal line (red) representing a proposed threshold. Boxes contain average and standard deviation of color intensity value per Ct group, where Neg is the group with undetectable RNA of SARS-CoV-2 by qRT-PCR.

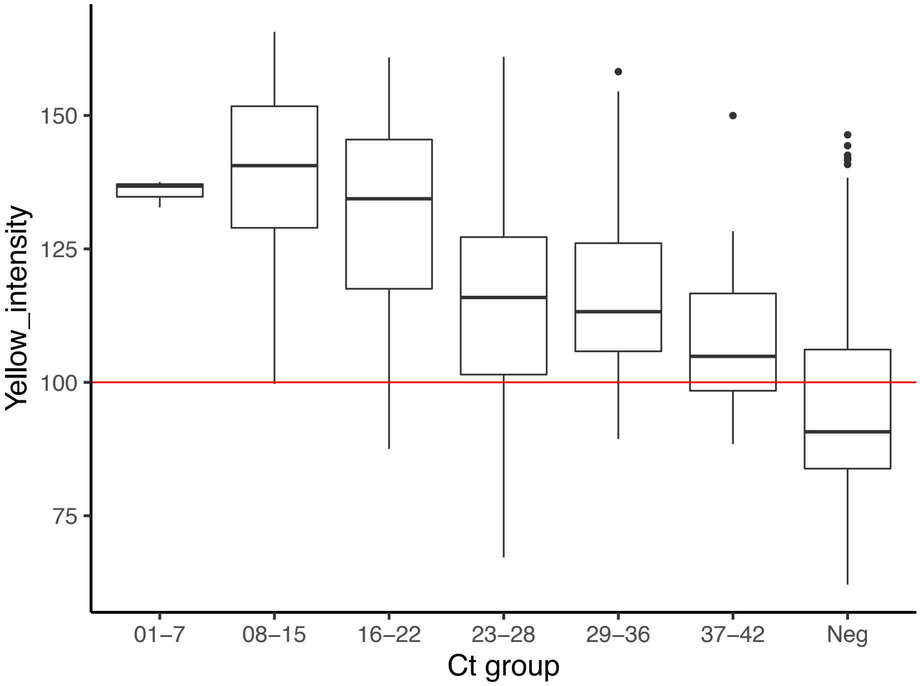

Supplement: Supplementary file 1 [file diagnostics-12-00848-s001.zip › diagnostics-1541070-supplementary/diagnostics-1541070-supplementary.pdf]
